# The Development of a Preference for Procedural Justice for Self and Others

**DOI:** 10.1038/s41598-018-36072-1

**Published:** 2018-12-10

**Authors:** Yarrow Dunham, Allison Durkin, Tom R. Tyler

**Affiliations:** 1 Yale University, Department of Psychology, New Haven, USA; 2 Yale University, School of Law, New Haven, USA

## Abstract

Adults prefer fair processes (“procedural justice”) over equal outcomes (“distributive justice”). This preference impacts their judgments of others in addition to their willingness to cooperate, raising questions about whether similar preferences drive judgments and behavior in children. The present study examines the development of this preference for procedural justice by testing children’s attitudes towards procedural justice using a resource allocation task in both first- and third-party contexts, and in contexts in which the procedurally just process does versus does not create distributional injustice. Results from children 4 to 8 years of age demonstrate that children robustly attend to and prefer procedural justice over distributive justice. However, younger children are less likely to prefer methods that are procedurally just or that create distributively just outcomes in first-party contexts, when distributive injustice might favor them. Results suggest an interplay between abstract justice concerns and the emerging ability to override selfishness.

## Introduction

A robust conception of fairness requires attention not only to outcomes but also to the *procedures* that produce those outcomes. Adults take both the outcome and the procedure into account in their evaluations of fairness^1–4^, and these judgments have wide-ranging consequences, influencing construal of everything from the basic allocation of resources to the estimation of political candidates and decisions to cooperate with police^5–7^. However, the developmental literature has generally focused on children’s judgments of the fairness of outcomes (*distributive justice*, i.e. the fairness associated with how resources end up being allocated) with much less attention to the fairness of the procedures by which those outcomes are produced (*procedural justice*, i.e. the fairness associated with processes by which resources are distributed).

The present study explores the children’s attitudes toward procedural justice using an experimental paradigm. A central focus of our inquiry is whether preferences for procedurally just procedures change based on two critical factors that have not yet been explored at length: whether the child him or herself stands to benefit from a less procedurally just procedure (placing justice concerns in conflict with self-interest), and whether the procedurally just procedure does versus does not create distributional injustice (placing two justice concerns in conflict with one another). In the remainder of this introduction we review past work on procedural justice in adults and children and then lay out our research questions in more detail.

## Procedural Justice Preference in Adults

Thibaut and Walker^1^ were the first to empirically study the psychology of procedural justice. Their work highlighted the importance of just procedures as sources for conflict resolution. Through a series of experiments, they found that satisfaction is not dependent solely on outcomes, but also on the decision-making procedures that shape these outcomes. Thibaut and Walker’s findings have been confirmed by numerous subsequent laboratory and field studies^2,3^. One striking illustration of the role of procedural justice is Tyler’s^8^ survey of defendants in Chicago, Illinois traffic court. Since police officers may not appear at traffic court hearings to explain the reasons for issuing tickets, and judges often consider coming to court and missing work sufficient punishment, defendants often have their cases dismissed without a hearing. If defendants were attending only to the outcome, they should be satisfied with this result. However, Tyler found that defendants that did not have a hearing often left the court unhappy, even if their tickets were dismissed. Tyler contends that this result is best explained by a model of outcome satisfaction that is grounded in procedural fairness: defendants felt that failing to receive a hearing made the process capricious, thus violating procedural justice.

## Development of a Distributive Justice Preference

It is expected that a preference for procedural justice would take time and experience with the world to learn. After all, understanding procedural justice would seem to require a wide range of antecedent cognitive skills as well as the ability to integrate them in sophisticated ways. This includes an understanding of cause and effect, a sense of fairness, and the understanding that different outcomes have different values for different individuals. A robust understanding of what processes are fair might also require understanding the concept of randomness, since some processes can be fair by virtue of being unpredictable and thereby not exploitable by involved parties^9,10^. More generally, children must also understand what makes a *process* fair. This requires evaluating situations by more than the outcome, and in some cases understanding that a process can be fair despite producing an unequal outcome. To arrive at a preference for fairness, in some particularly critical cases children must also override their own selfishness and come to prefer processes that are procedurally fair even when that process might materially disadvantage them. It stands to reason that children may initially learn and prefer a simpler distributive logic, that of distributive justice with its emphasis on equal outcomes. Below, we review the relevant existing research on distributive fairness in children.

Research examining the development of children’s perceptions of the fairness of outcomes is diverse and abundant. Attentiveness to the fairness of outcomes emerges early in development, with children expecting equal resource distribution among third parties even in infancy^11,12^. Geraci and Surian^13^ also demonstrated that infants exhibit a significant preference for individuals who enact equal distributions. By 3-years of age, children direct a protagonist to distribute resources equally among third parties, regardless of the relationship of the protagonist to the other parties, indicating knowledge that they should enact distributive justice^14^. This preference for equal outcomes is also known as *inequity aversion*^15–17^.

Inequity aversion in the context of third-party distributions appears to be consistent with *disadvantageous inequity aversion* in a first-person context. Disadvantageous inequality aversion refers to a desire not to receive less than another^18^. Children ages 4–10 reject unequal distributions both in third-party cases and in cases in which the child herself would be a disadvantaged recipient^19,20^. Children frequently even choose to throw a resource away rather than distributing it if the distribution would create inequality^21^. However, the possibility that disadvantageous inequity aversion is driven by self-interest rather than fairness is suggested by cross-cultural and developmental work that also examines *advantageous inequity aversion* (a preference not to receive more than another). In one large-scale study, disadvantageous inequity aversion consistently emerged by middle childhood, while advantageous inequity aversion emerged later and in a more culturally contingent manner, and was wholly absent in some cultural settings^22^.

The present study focuses on the emergence of justice orientation in children in the United States, and given that the bulk of the developmental literature focuses on this population we also focus the remainder of our review there, though to the best of our knowledge a similar conclusion could be reached with a focus on other Western countries. In the US context, advantageous inequity aversion emerges between 8–10 years and grows stronger with age^22^, while children younger than 8 are considerably more likely to accept unequal distributions when they are the advantaged party^19^. Indeed, younger children (3–4-year-olds) will actively distribute resources to themselves so that they are at an advantage^23^, and will take a cost in order to remain at a *relative* advantage even when the alternative is to get more overall. For example, children ages 5–6 preferred to give themselves seven tokens and a third party zero tokens, rather than give eight tokens to both themselves and a third party^20^.

Many researchers have contended that acting fairly is beneficial because it improves future reciprocity and generosity^2,23,24^. However, Shaw^25^ posits that fairness may instead be a consequence of a desire to appear impartial. Evidence for this account comes from children’s greater willingness to treat others unfairly when their unfair behavior is anonymous^26^. Further, older children are more likely to create disadvantageous than advantageous inequity, presumably because the former can be considered a way of avoiding rather than giving vent to partiality^27^. The impartiality account offers a natural transition from issues of distributional justice to issues of procedural justice, because procedurally just processes sometimes create distributional injustice (i.e., they sometimes create unequal outcomes), but they do so through an impartial procedure. How and when does an appreciation of procedural justice emerge?

## Development of a Procedural Justice Preference

The majority of research addressing procedural justice understanding in children has focused on legal socialization in adolescents, i.e. the process by which children develop an understanding of and compliance with the law^28,29^. This work has suggested that satisfaction with hypothetical legal procedures depends both on the outcome and the presence of fair and impartial procedures^30–32^, a pattern that at least one study observed in elementary school children^33^. However, most of this work has used verbal responses to hypothetical scenarios, raising questions concerning how they map onto children’s actual behavior. More recently, a few researchers have examined children’s understanding of procedural justice distinct from its legal implications. In a study whose design is particularly relevant to the present research, researchers used either a fair or an unfair spinner to distribute stickers to triads of preschool-aged children^34^. Experimenters placed either the fair or unfair spinner in the room, and children, without an experimenter present but in the presence of their peers, were able to spin it to determine how many stickers each student should receive. Over the course of the experiment, researchers found that children were less likely to object to distributive unfairness (i.e. unequal outcomes) if the fair spinner was used.

In a study using a related design, Shaw and Olson^35^ asked for the judgments of children ages 5–8 as to whether an extra sticker should be distributed to two third parties using a spinner, or whether the sticker should be thrown away. The relative partiality of the spinner varied across conditions. The alternatives were (1) both third parties were represented equally on the spinner, constituting a procedurally just procedure because no party was disadvantaged, (2) one third party had greater representation on the spinner, constituting a procedurally unjust procedure because one party was unfairly advantaged over the other, and (3) one third party represented the entire spinner, constituting an even more procedurally unjust procedure. Shaw and Olson found that children of all ages preferred spinning an impartial wheel, a fair procedure which resulted in inequality of outcome, to throwing the resource way, which preserved the equality of outcome. The older children preferred throwing the resource away to spinning a biased wheel, while the younger children did not show a preference between the biased wheel and throwing the resource away. Shaw and Olson posit that children ages 5 and older prefer not to waste a resource when an impartial procedure is available, but younger children were more willing to use a partial procedure in general.

## Present Study

The current study examined the extent of children’s attention to procedural justice using resource allocation tasks closely modeled after work by Shaw and Olson^35^. We add to this work by examining what happens when procedural and distributional concerns are in conflict by manipulating two critical factors. First, we contrast a procedurally fair way of allocating an extra resource, but one that produces inequality (flipping a coin) with two different alternative processes: (1) a process that could be considered procedurally just but which does not produce inequality, though it wastes a resource (throwing the resource away), and (2) a partial process that produces inequality (giving the resource directly to one party). Second, we examine children’s choices between these options in both first-party contexts, in which their own outcomes are directly affected by their choices, and third-party contexts, in which their own outcomes are unaffected. Following past work we used consequential real decisions rather than attitudes toward vignettes, such that children could keep any resources they allocated to themselves. Thus, in some cases invoking a procedurally just process could be costly to the participant. Because of concerns that younger children might not understand the randomness of a spinner or how its partiality can be manipulated^36–38^, we used a coin flip procedure instead, including an extended training period with the coin to maximize the chances that its characteristics would be understood.

We were motivated by three primary hypotheses: (1) Among two procedurally fair procedures, children ages four and up will prefer to conserve a resource even if it results in distributive unfairness, replicating a pattern of results suggested by Shaw and Olson^35^. (2) When two procedures result in distributively unfair outcomes, children who do not have a stake in the outcome will prefer the fair procedure to the unfair procedure. (3) When children do have a stake in the outcome, younger but not older children will prefer an unfair procedure that advantages them to a fair procedure that does not. Thus, we predicted a robust preference for procedural justice, but one that appears in third-party contexts prior to first-party contexts.

## Method

### Participants

One hundred and twelve children ages 4 to 10 (*M*_age_ = 80.7 months; *SD*_age_ = 14.06 months; 59 girls) attending preschool through second grade completed the study. These included five 4-year-olds, twenty-five 5-year-olds, thirty-eight 6-year-olds, twenty-two 7 year-olds, twenty 8-year-olds, and one 10-year-old. Participants were recruited from private religious schools in the Northeast area of Pennsylvania and were predominantly white. Four participants were excluded from the final analysis (two due to missing data, one due to incompletion of the experiment, and one for falling outside of the relevant age range of 4–8 years), leading to a final sample of one hundred and eight. This project was reviewed and approved by the Yale Human Subjects Committee and was conducted in accordance with that approval.

### Design and procedure

**Figure 1:**
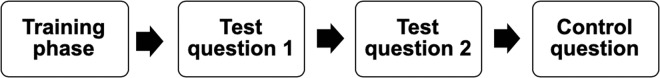
Progression of the experiment for each participant.

Informed consent was secured in writing from parents or legal guardians and child verbal assent was secured prior to testing. All participants were tested individually during the school day in rooms located proximate to school classrooms. Each participant took approximately 5 minutes to complete the study. The study employed a 2 (within-subject: first vs. third person) X 2 (partial within/between-subject: whether the alternative to flipping the coin was to give versus throw away the resource) mixed design. Each child answered two of the four test questions preceded by an additional training phase at the start and followed by a standard control question at the end, as shown in Fig. 1.

Following Grocke and colleagues^34^, a training phase was employed in order to give children familiarity with the fair procedure and the unpredictability of its outcome, absent the choice of how to allocate resources. The fair procedure was to flip a coin-like object that was red on one side and blue on the other, hereafter referred to as the coin. During the training phase, the experimenter showed the coin to the participant and demonstrated flipping the coin six times. After each flip, participants were instructed to distribute a resource to one of two colored “paper monsters,” according to which side of the coin landed facing upward (see Appendix A). Participants were asked before and after the training phase whether they could predict the outcome of the coin flip in order to promote understanding of the coin flip’s unpredictability; participants’ answers were neither validated nor corrected.

Test and control scenarios were adapted from Shaw and Olson^35^ and were resource allocation tasks in which children were asked what to do with an extra resource. Children were presented with two options and answers were forced choice. Consistent with previous research, stickers were used as the resource. To ensure that the children were interested in the sticker, each child was asked to choose her favorite kind of sticker from among five options. The chosen type of sticker was selected from the other stickers and used throughout the remainder of the experiment. Children were allowed to keep any stickers that they earned during the experiment.

Across all test questions, there were two recipients of the allocation; the identities of the recipients varied across questions. The two recipients were either two third parties unknown to the participant (third-person scenario), or one unknown third party and the participant herself (first-person scenario). The pairs of choices presented to children regarding how to allocate the resource also varied. One option always available to participants was to flip the coin (procedurally just but distributively unjust) to determine who received the resource. The second option was *either* to throw the extra resource away (procedurally and distributively just), or to give the extra resource to a recipient specified by the experimenter; this was a randomly selected third party in the third-party case, or the participant him or herself in the first-party case (procedurally and distributively unjust). The four test questions are depicted in Table 1.

**Table 1:** The Four Possible Test Questions Across Conditions.

Person	Test questions	
	Flip coin or give away resource	Flip coin or throw away resource
1^st^	Flip or give to self	Flip or throw away
3^rd^	Flip or give to third party	Flip or throw away

Participants always answered one first-person and one third-person question with question type and question order counterbalanced, creating eight possible orderings.

Following the test questions participants answered a control question that was consistent across participants. It was presented last in order to assure that it did not impact test questions. In the control condition, one recipient had more resources at the outset than the other recipient (two compared to one). Children had a choice between giving a resource to a disadvantaged third party, and flipping a coin to determine whether the advantaged or disadvantaged third party would receive the resource. Here, flipping the coin could be viewed as partial, since participants are foregoing the sure opportunity to rectify the inequality to instead take a gamble that could either rectify or exacerbate the inequality^35^. Consequently, it was predicted that if children were concerned with justice, children should choose to give the sticker to the disadvantaged party rather than flipping the coin, which could produce even greater distributive unfairness. Thus, this condition allowed us to ensure that children did not rigidly consider flipping the coin to be the correct outcome irrespective of the distributional context. Across all conditions, after a child answered a test question the chosen action was always performed for the child and the stickers distributed according to the result of the chosen procedure.

### Analysis strategy

Because all primary outcomes were dichotomous choices between two ways of distributing a resource, we used logistic regression predicting the choice to flip the coin. In the test condition predictors were age, gender, whether the task was first- or third-person, and whether the alternative to flipping the coin was throwing away the sticker or unfairly distributing it to one party. In the control task predictors were age and gender. We tested for main effects and interactions, retaining terms when a likelihood ratio test (LRT) suggested that they improved model fit or where they figured in higher-order interactions. Where within-participant factors are present we use mixed models with choices nested within participants. In all cases we present regression coefficients and 95% confidence intervals for retained effects, and conduct comparisons to chance via binomial tests. For ease of presentation participants were divided at the median into a younger age group (*M*_age_ = 69.6 months; *SD*_age_ = 7.27 months; 29 girls) and an older age group (*M*_age_ = 91.3 months; *SD*_age_ = 7.64 months; 29 girls), but results with age treated as a continuous variable were qualitatively similar and do not substantively change any reported result. Data and an analysis script to reproduce results and figures reported here are available online at https://osf.io/cdx67/.

## Results

### Control condition

**Figure 2:**
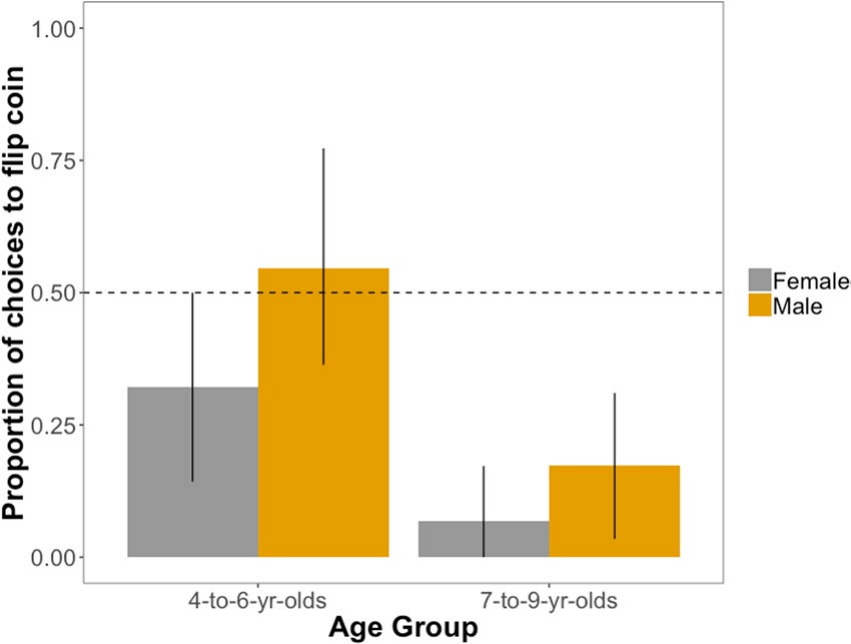
Observed proportion of choices to flip the coin (versus rectify the inequality) as a function of participant age group and gender in the control condition in which one party initially had 2 stickers while the other party had 1 sticker. Error bars are bootstrapped 95% confidence intervals; dashed horizontal line represents chance responding.

While the control condition was presented last, we report it first here because if children do not seek to rectify clear inequalities toward a third person then their behavior in other conditions would be difficult to interpret. Participants were presented with an unequal distribution between two third parties in which one party had 2 stickers and the other party had 1 sticker. The participant then chose between flipping the coin to determine who would get an additional sticker or distributing it directly to the disadvantaged party. Overall 87.9% of the older age group chose to give a resource to the disadvantaged party rather than flip the coin, compared to 58.0% of the younger age group. This main effect of age was significant, with older participants less likely to flip the coin (*β* = −1.79, CI = [−2.80; −0.79], Odds Ratio (OR) = 0.17, *p* = 0.0005). There was also an unexpected main effect of gender, with boys more likely to flip the coin than girls (*β* = 0.96, CI = [0.00; 1.92], OR = 2.62, *p* = 0.049); see Fig. 2. Comparing choices for each age group to chance via a binomial test, older children (*p* < 0.001) preferred to rectify the inequality by giving a sticker to the disadvantaged party, while younger children did not show a clear preference (*p* = 0.32). Thus, older children consistently sought to rectify an inequality while younger children, especially younger boys, were more likely to flip the coin, a choice that could either rectify or exacerbate the inequality.

### Test conditions

Recall that in the test conditions the two parties have an equal endowment of one sticker and the participant determines the procedure that will allocate (or not allocate) a third sticker. All terms involving gender failed to add to model fit and so were dropped. The final minimal model included a random intercept for participant, a main effect of age group, indicating that older children were less likely to flip the coin (*β* = −1.11, CI = [−2.06; −0.16], *p* = 0.022), as well as two interactions, one of which qualified this main effect. The first interaction was between person (first or third) and the alternative option (throw away or give unfairly) (*β* = 2.67, CI = [1.00; 4.34], *p* = 0.002), demonstrating that choosing the alternative to flipping the coin varied more in the third-person than in the first-person condition. This effect must also be interpreted in light of the second interaction, between age group and the alternative option (*β* = 2.29, CI = [0.74; 3.85], *p* = 0.004), demonstrating that use of the two options varied more for older than younger children. To unpack these interactions we conducted follow-up analyses separately across the within-subjects factor of person (first or third), resulting in two independent logistic regressions for each of those conditions.

**Figure 3:**
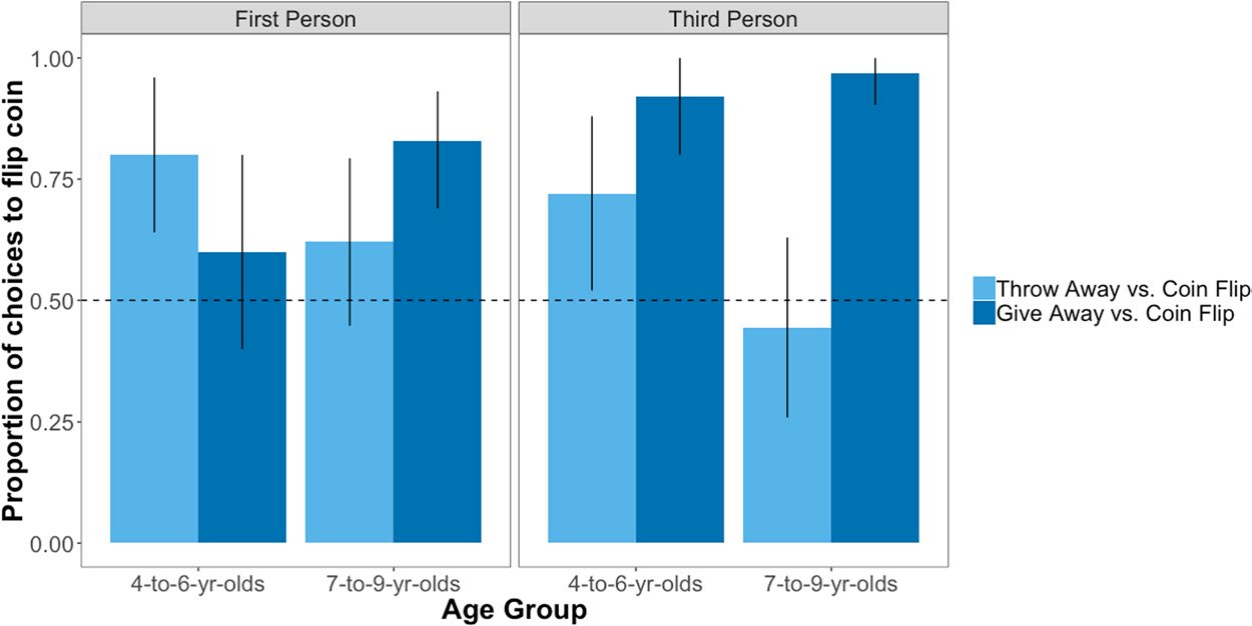
Observed proportion of choices to flip the coin as a function of age group and alternative (throw away or unfairly give) in the first-person (left panel) and third-person (right panel) test conditions. Error bars are bootstrapped 95% confidence intervals; dashed horizontal line represents chance responding.

Beginning with first-person contexts (Fig. 3, left panel), choices to flip the coin were influenced by an interaction between the available alternative and age group, (*β* = 2.06, CI = [0.30; 3.82], *p* = 0.022); younger children were 2.67 times more likely to choose to flip the coin when the alternative was to throw away the resource (80% of trials, binomial *p* = 0.004) than when it was to get the resource themselves (60% of trials, *p* = 0.44). By contrast, older children were 2.93 times more likely to choose to flip the coin when the alternative was to get the resource themselves (83% of trials, *p* = 0.005) than when it was to throw the resource away (62% of trials; *p* = 0.26).

Turning to the third-party condition (Fig. 3, right panel), analysis revealed a sole main effect of the available alternative (*β* = 2.56, CI = [1.27; 3.85], *p* < 0.001); participants of both age groups were more likely (OR = 12.96) to choose to flip the coin when the alternative was to give the resource to one party than when it was to throw away the resource. Binomial tests suggested that younger participants preferred to flip the coin both when the alternative was to throw the resource away (*p* = 0.04) and when the alternative was to unfairly distribute it (*p* < 0.001), while older children only preferred to the flip the coin when the alternative was the unfair distribution (*p* < 0.001) and not when it was to throw it away (*p* = 0.70), though these follow-up analyses should be interpreted cautiously given a lack of interaction between age group and alternative when considering the third-person condition separately.

## Discussion

We begin our discussion by reviewing our three primary predictions. First, our prediction that children would prefer a procedurally just process that conserves rather than destroys a resource was supported for younger children but not older children; younger children preferred to flip a coin to distribute an extra resource, even though it resulted in distributional inequality, while older children appeared indifferent between these two options. This result held across first- and third-person outcomes, suggesting it is not merely a result of the fact that in the first-party case flipping the coin provides an opportunity for the participant herself to gain a resource. Rather, younger children appeared more motivated to avoid the destruction of a resource, even though that produced distributional inequality. Older children did not generally show that pattern, perhaps because they perceived merit in both procedures.

We also predicted that children without a stake in the outcome (i.e. in the third-party case) would more strongly prefer the procedurally just process; this prediction was supported for both younger and older children, who almost never advocated giving the resource out in a partial manner when an impartial procedure (flipping the coin) was available. Finally, we predicted that this pattern would break down in one specific case, namely for younger children in a first-party case in which the partial allocation would directly benefit them. This prediction was largely supported: younger children in the first-party flip-or-give condition were the only group to not robustly prefer to flip the coin, though they also did not, as a group, prefer to receive the resource overall. Nonetheless, the chance responding in this condition can be interpreted as evidence of conflict between self-interest and impartiality, a conflict that appeared to be readily resolved by older children as well as younger children in third-party contexts. One plausible reason for this shift is the protracted development of advantageous inequity aversion observed in past work^22^. Indeed, even in adults avoiding advantageous inequities appears to take greater cognitive resources, consistent with the idea that it is a more laboriously acquired norm^39^. Thus, our results dovetail with past work^34,35^ suggesting that even preschool age children do appreciate some aspects of procedural justice, and choose procedurally just outcomes as long as they are not in conflict with self-interest.

The results of this research are largely consistent with the impartiality account of fairness^21,25,35^. According to the impartiality account, distributive unfairness is only truly unfair if it is a result of a form of partiality. Children, even younger children, in third-party contexts robustly chose an impartial method (flipping a coin) over a partial method (giving out the resource) even when both produced distributive unfairness. Indeed, even when children could have given the resource to themselves, neither age group ever demonstrated a preference for the partial procedure and older children robustly preferred the impartial procedure. Our findings can also be placed in the context of studies showing other conditions under which children depart from the norm of distributive justice, for example when one person deserves a majority allocation^40^.

This study did not replicate Shaw and Olson’s^35^ finding that children preferred to use a fair procedure (a spinner) over throwing a resource away. Instead, it was found that children performed at chance between these two options in the most relevantly comparable third-party cases. While this result was unanticipated, it could occur if children understood the randomness of the coin better than the spinner, either from the start or as a consequence of our training procedure.

Unlike past research employing a very similar procedure^35^, we observed an unexpected effect of gender in our control condition in which children chose between distributing an extra resource to rectify a pre-existing inequality and flipping a coin to distribute the extra resource, with the risk of exacerbating the inequality. Across age groups boys were more likely to choose to flip the coin. Because it diverges from past findings and was unexpected, and because gender did not impact our test conditions, we do not offer strong interpretations here. However, it is possible that boys had a stronger or more rigid desire to employ the procedure that appeared more procedurally just even in a context in which that procedure in fact had a high probability of leading to a seemingly unfair outcome. The possibility that boys at these ages are less flexible in their application of principles of procedural justice could be investigated in future work.

It is possible that placing the control condition last unduly impacted the results of that question. Children may have begun choosing to give the sticker to a participant rather than flipping the coin to decide who received it because after having flipped the coin several times they were no longer as interested in exploring this procedure. A related possibility is that children were familiar by this point with how the resources were typically distributed among participants (two for each participant, receiving one by one in alternation), and may have chosen to give the resource to the disadvantaged participant in order to complete a perceived pattern. Future research would need to be conducted with a larger sample size in order to conclusively confirm that order had no effect on control condition results.

Of note is that children were asked how *should* we allocate than how they *wanted* to allocate. This has proven to be a meaningful distinction for children in other contexts^41,42^. The use of *should* was useful in the present context, which sought to examine children’s emerging understanding of justice and how children employ justice-related principles. However, future research could examine what effect this wording has on children’s valuation of procedural justice, testing the possibility that asking children how they want to distribute would produce more self-serving behavior. Still, it is important to note that children clearly understood that their response to the *should* question would determine how resources were actually allocated, so we do not think this wording had a major influence on results.

We also note that there are many ways to operationalize procedural justice, and the ways we chose here are not without limitation. Consider throwing a resource away. It is possible that children (as well as some readers) may not share the intuition that this is a form of procedural justice. At the very least destroying a resource can be considered an economically inefficient means of ensuring equality, one that some children may have avoided for reasons other than an interest in procedural justice. Further, flipping a coin to allocate a resource does not necessarily instantiate a clear ethical principle such as merit. Nonetheless, both of these procedures do meet the core criteria of impartiality that we used as the basis of our inquiry^25^, and in our experience both are procedures that children themselves sometimes spontaneously employ as a means of achieving satisfactory outcomes among themselves. Further, both procedures would likely be considered procedurally just on influential philosophical accounts because, while we may lack an independent procedure to determine the fairest possible outcome, we do have a procedure which can be agreed upon and which is itself impartial, what Rawls called *pure* procedural justice^43^. Thus, we believe these are reasonable forms of procedural justice, though research on alternative operationalizations would be useful.

Given the potential role of the appearance of impartiality in guiding judgments, and the competing account of fairness as promoting generosity and reciprocity, future research might also remove the experimenter from children’s eyesight, as in one past study^34^. On Shaw’s^25^ impartiality account, this might lead to more self-serving behavior. Other interesting avenues include varying whether or how well children know the recipients of the allocation, whether the recipients will learn of the participant’s allocations, and whether the participant and the recipients of the allocation will cooperate in the future. Additionally, the current study examined children’s attitudes toward procedural justice when in an arbitrator’s role; that is, they were given the choice of what method to use. Future research could examine children’s perceptions of others who employ fair or unfair methods that result in equal or unequal outcomes.

This study adds to the emerging field of research examining children’s developing understanding of procedural justice. The results demonstrate that children have a robust preference for procedural justice, even when a procedurally just process leads to distributive inequality. It also demonstrates that over early childhood children begin to embrace procedural justice even when their own outcomes might be harmed by just strategies, though doing so appears difficult for younger children. While this represents just one small slice of research on the broader process of moral and legal socialization, it suggests that with age children show an increasing concern with fair processes and the general appearance of impartiality in how resources are distributed.

## Electronic supplementary material

Supplemental Online Materials

## References

[1] Thibaut, J., & Walker, L. *Procedural Justice: A Psychological Analysis*. Hillsdale, N.J.: Lawrence Erlbaum Associates (1975).

[2] Lind E. Allan, Tyler Tom R. (1988). Introduction. The Social Psychology of Procedural Justice.

[3] Tyler TR (2000). Social justice: Outcome and procedure. Int. J. Psychol..

[4] Tyler, T. R. *Why People Obey the Law: Procedural Justice, Legitimacy, and Compliance*. Princeton, NJ: Princeton Univ (2006).

[5] Tyler TR, Rasinski K, Spodick N (1985). The influence of voice on satisfaction with leaders: Exploring the meaning of process control. J. Pers. Soc. Psychol..

[6] Sunshine J, Tyler TR (2003). The role of procedural justice and legitimacy in shaping public support for policing. Law. Soc. Rev..

[7] Tyler TR, Fagan J, Geller A (2014). Street stops and police legitimacy: Teachable moments in young urban men’s legal socialization. J. Empirical Legal Stud..

[8] Tyler TR (1988). What is procedural justice?: Criteria used by citizens to assess the fairness of legal procedures. Law. Soc. Rev..

[9] Choshen-Hillel S, Shaw A, Caruso EM (2015). Waste management: How reducing partiality can promote efficient resource allocation. J. Pers. Soc. Psychol..

[10] Keren G, Teigen KH (2010). Decisions by coin toss: Inappropriate but fair. Judgm. Decis. Mak..

[11] Schmidt MF, Sommerville JA (2011). Fairness expectations and altruistic sharing in 15-month-old human infants. PloS one.

[12] Sloane S, Baillargeon R, Premack D (2012). Do infants have a sense of fairness?. Psychol. Sci..

[13] Geraci A, Surian L (2011). The developmental roots of fairness: Infants’ reactions to equal and unequal distributions of resources. Dev. Sci..

[14] Olson KR, Spelke ES (2008). Foundations of cooperation in young children. Cognition.

[15] Baumard N, Mascaro O, Chevallier C (2012). Preschoolers are able to take merit into account when distributing goods. Dev. Psychol..

[16] Hook J, Cook TD (1979). Equity Theory and the Cognitive Ability of Children. Psychol. Bull..

[17] Sigelman CK, Waitzman KA (1991). The development of distributive justice orientations: contextual influences on children’s resource allocations. Child. Dev..

[18] Fehr E, Schmidt KM (1999). A theory of fairness, competition, and cooperation. Q. J. Econ..

[19] Blake PR, McAuliffe K (2011). “I had so much it didn’t seem fair”: Eight-year-olds reject two forms of inequity. Cognition.

[20] Sheskin M, Bloom P, Wynn K (2014). Anti-equality: Social comparison in young children. Cognition.

[21] Shaw A, Olson KR (2012). Children discard a resource to avoid inequity. J. Exp. Psychol. Gen..

[22] Blake PR (2015). The ontogeny of fairness in seven societies. Nature.

[23] Fehr E, Bernhard H, Rockenbach B (2008). Egalitarianism in young children. Nature.

[24] Gintis H, Henrich J, Bowles S, Boyd R, Fehr E (2008). Strong reciprocity and the roots of human morality. Social Justice Research.

[25] Shaw A (2013). Beyond “to share or not to share” The impartiality account of fairness. Curr. Dir. Psychol. Sci..

[26] Shaw A (2014). Children develop a veil of fairness. J. Exp. Psychol. Gen..

[27] Shaw A, Choshen-Hillel S, Caruso EM (2016). The development of inequity aversion: understanding when (and why) people give others the bigger piece of the pie. Psychol. Sci..

[28] Easton, D., & Dennis, J. *Children in the political system: The origins of political legitimacy*. Chicago: University of Chicago Press (1969).

[29] Fagan J, Tyler TR (2005). Legal socialization of children and adolescents. Social Justice Research.

[30] Cashmore, J. Children’s perceptions of Children’s Court outcomes in welfare and criminal matters. Paper presented at the Fifth Australian Developmental Conference, Sydney, Australia (1988).

[31] Hicks AJ, Lawrence JA (1993). Children’s criteria for procedural justice: Developing a young people’s procedural justice scale. Social Justice Research.

[32] Tyler TR, Rasinski K, Spodick N (1985). The influence of voice on satisfaction with leaders: Exploring the meaning of process control. J. Pers. Soc. Psychol..

[33] Gold LT, Darley JM, Hilton JL, Zanna MP (1984). Children’s perceptions of procedural justice. Child. Dev..

[34] Grocke P, Rossano F, Tomasello M (2015). Procedural justice in children: Preschoolers accept unequal resource distributions if the procedure provides equal opportunities. J. Exp. Child. Psychol..

[35] Shaw A, Olson K (2014). Fairness as partiality aversion: The development of procedural justice. J. Exp. Psychol..

[36] Metz KE (1998). Emergent understanding and attribution of randomness: Comparative analysis of the reasoning of primary grade children and undergraduates. Cogn. Instr..

[37] Piaget, J., & Inhelder, B. *The origin of the idea of chance in children*. London: Routledge & Kegan Paul (1975).

[38] Kuzmak SD, Gelman R (1986). Young children’s understanding of random phenomena. Child. Dev..

[39] van den Bos, Peters SL, Bobocel DR, Ybema JF (2006). On Preferences and Doing the Right Thing: Satisfaction with Advantageous Inequity When Cognitive Processing Is Limited. J. Exp. Social Psychol..

[40] Schmidt MF, Svetlova M, Johe J, Tomasello M (2016). Children’s developing understanding of legitimate reasons for allocating resources unequally. Cogn. Dev..

[41] Smith, C. E., Blake, P. R., & Harris, P. L. I should but I won’t: Why young children endorse norms of fair sharing but do not follow them. *PloS ONE*, **8**(8), 10.1371/annotation/4b9340db-455b-4e0d-86e5-b6783747111f (2013). 10.1371/journal.pone.0059510 PMC3603928 23527210

[42] Sheskin M (2016). Some equalities are more equal than others: quality equality emerges later than numerical equality. Child. Dev..

[43] Rawls, J. *A Theory of Justice*. Cambridge: Harvard University Press (1971).

