# Supplementary material for: The Development of a Preference for Procedural Justice for Self and Others
Source: Sci Rep. 2018 Dec 10;8:17740. doi: 10.1038/s41598-018-36072-1 (PMC6288281; doi:10.1038/s41598-018-36072-1)
Supplement: Supplementary file 1 — Supplemental Online Materials [file 41598_2018_36072_MOESM1_ESM.pdf]

The Development of a Preference for Procedural Justice for Self and Others

Yarrow Dunham[a*], Allison Durkin[b], and Tom Tyler[c]

[a] Yale University, Department of Psychology

[b] Icahn School of Medicine at Mount Sinai, Department of Psychiatry

[c] Yale University, School of Law

Corresponding author:

Yarrow Dunham, yarrow.dunham@yale.edu

Supplemental Online Materials (Appendix A)

Vignettes

Training Phase

Training instructions for all participants. "We're going to play a game. Before we start the game, I need help feeding some monsters. Can you help me do that? To feed the monsters we're going to use this [coin]. There's a different color on each side. If I throw this in the air, can you tell before it hits the table what color it's going to land on? Now we're going to feed the monsters. I'm going to throw this in the air. If it lands on this side [show red side of coin] can you feed the red monster, and if it lands on this side [show blue side of coin] can you feed the blue monster? [Give participant 6 pieces of "food." Flip coin 6X and allow child to give out food, pointing out and correcting mistakes]. Thanks! When I threw this in the air, did you know before it hit the table what color this was going to land on?"

Test Questions

Test instructions for all participants. "Now we're going to play the game. I'm going to ask you some questions–there are no right or wrong answers, I just want to know what you think!"

Gender in all vignettes. The gender of the third party recipient was always matched to that of the participant, so that the third-party for male participants was Tom, and Anne for female participants.

First person condition vignette: flip or give to self / flip or give to third party. "[Two envelopes, one blank and one with the letter T/A on it, are placed facing children.] You and another student did a great job helping me, so I want to give you both stickers as a reward. I have five stickers. I have one for Tom/Anne, and one for you. One for Tom/Anne, and one for you.

[One sticker is placed on Tom/Anne's or the participant's corresponding envelope during each use of "one for."] Uh oh! I have one left over. What should we do? We can either flip the coin and if it lands on red we'll give it to Tom/Anne and if it lands on blue we'll give it to you, or I can give the sticker to you/throw the sticker away and no one will get it. What should we do?"

Third person condition vignette: flip or give to third party / flip or throw away. "[Two envelopes, one with the letter M and one with the letter D on it, are placed facing children]. Earlier today, two kids from another school named Mark and Dan did a great job cleaning up their rooms, and I want to give them stickers as a reward. I have five stickers. I have one for Mark, and one for Dan. One for Mark, and one for Dan. [One sticker is placed on Mark's or Dan's corresponding envelope during each use of "one for."] Uh oh! I have one left over. What should we do? We can either flip the coin and if it lands on red we'll give it to Mark and if it lands on blue we'll give it to Dan, or I can give the sticker to Dan/throw the sticker away and no one will get it. What should we do?"

Control Question

Control question vignette for all participants. "I just have a few more questions. Okay? [Two envelopes, one with the letter K and one with the letter G, are placed facing children]. Earlier today, two kids from another school named Kate and Grace did a great job doing their homework, and I want to give them stickers as a prize. I have four stickers. I have one for Kate, and one for Grace. One for Kate. [One sticker is placed on Kate or Grace's corresponding envelope during each use of "one for."] Uh oh! I have one left over. What should we do? We can either flip the coin and if it lands on red we'll give it to Kate and if it lands on blue we'll give it to Grace, or I can give the sticker to Grace. What should we do?"
